# Health Benefits of *Lactobacillus gasseri* CP2305 Tablets in Young Adults Exposed to Chronic Stress: A Randomized, Double-Blind, Placebo-Controlled Study

**DOI:** 10.3390/nu11081859

**Published:** 2019-08-10

**Authors:** Kensei Nishida, Daisuke Sawada, Yuki Kuwano, Hiroki Tanaka, Kazuhito Rokutan

**Affiliations:** 1 Department of Pathophysiology, Institute of Biomedical Sciences, Tokushima University Graduate School, Tokushima, Tokushima 770-8503, Japan; 2 Core Technology Laboratories, Asahi Quality & Innovations, Ltd., 11-10, 5 Chome, Fuchinobe, Chuo-ku, Sagamihara-shi, Kanagawa 252-0206, Japan

**Keywords:** heat-inactivated *Lactobacillus gasseri* CP2305, psychobiotics, healthy young adults, stress, mental health, sleep quality, fecal microbiota

## Abstract

Short-term administration of *Lactobacillus gasseri* CP2305 improves stress-associated symptoms and clinical symptoms in healthy young adults and in patients with irritable bowel syndrome, respectively. We evaluated the efficacy and health benefits of the long-term use of a tablet containing heat-inactivated, washed *Lactobacillus gasseri* CP2305 (CP2305) in healthy young adults. Sixty Japanese medical students (41 men and 19 women) preparing for the national examination for medical practitioners ingested CP2305-containing or placebo tablets once daily for 24 weeks. Intake of the CP2305 tablet significantly reduced anxiety and sleep disturbance relative to placebo, as quantitated by the Spielberger State-Trait Anxiety Inventory and the Pittsburgh Sleep Quality Index. Single-channel sleep electroencephalograms show that CP2305 significantly shortened sleep latency and wake time after sleep onset and increased the delta power ratio in the first sleep cycle. CP2305 also significantly lowered salivary chromogranin A levels compared with placebo. Furthermore, 16S rRNA gene sequencing of participant feces demonstrated that CP2305 administration attenuated the stress-induced decline of *Bifidobacterium* spp. and the stress-induced elevation of *Streptococcus* spp. We conclude that the long-term use of CP2305-containing tablets may improve the mental state, sleep quality, and gut microbiota of healthy adults under stressful conditions.

## 1. Introduction

The bidirectional communication system between the gut and brain, the gut–brain axis, has been shown to have a crucial role in the maintenance of intestinal homeostasis and brain function [1,2]. The microbial community of the gastrointestinal tract affects this communication system through immune, endocrine, and neural pathways [3]. Indeed, several lines of evidence suggest that the gut microbiome has a significant impact on brain function, affecting mood, recognition, and behavior [4]. Thus, the concept of the gut–brain axis has expanded to the “microbiota–gut–brain axis” [4].

Probiotics, defined as live microorganisms which confer a health benefit to the host when administered in adequate amounts [5], have also been shown to interact with the brain. In particular, animal studies have reported that probiotic administration modulates the hippocampus-mediated negative feedback regulation of the hypothalamic–pituitary–adrenal (HPA) axis and mitigates stress-induced visceral pain and behavior [6,7]. Probiotics have also been shown to transduce signals to the brain via the afferent vagal nerve and relieve mood disturbances [8,9]. Accordingly, the term “psychobiotics” refers to live microbes that confer a positive mental health benefit [10]. In human trials, the probiotic *Lactobacillus plantarum* 299v complemented treatment with selective serotonin reuptake inhibitors, improving the cognitive performance of patients with major depressive disorder [11]. Similarly, administration of *Bifidobacterium longum* NCC3001 reduced depression, but not anxiety scores, and improved the quality of life in patients with irritable bowel syndrome (IBS). These observations were shown to be associated with reduction of the amygdala and fronto-limbic reactivity [12]. *Lactobacillus casei* strain Shirota has also been reported to relieve stress-associated symptoms in young adults experiencing stressful situations [9].

In addition to live bacteria, heat-inactivated probiotics have been shown to exert beneficial effects. For example, administration of heat-killed *Lactobacillus acidophilus* L-92 reduced symptoms of atopic dermatitis in adults [13]. In hamsters, supplementation of a high-fat diet with heat-killed *Lactobacillus reuteri* GMNL-263 mitigated fatty liver syndrome and fibrosis of the liver and heart through a reduction in the expression of transforming growth factor β [14]. Wei et al. [15] showed that both live and heat-killed *Lactobacillus paracasei* PS23 reversed chronic corticosterone-induced anxiety- and depression-like behaviors via prevention of the corticosterone-induced decline of brain-derived neurotrophic factor, mineralocorticoid, and glucocorticoid receptor levels in the hippocampus.

Similarly, *Lactobacillus gasseri* CP2305, which was originally isolated from the stool of a healthy volunteer and colonizes the digestive tract of 40% of recipients [16], has been shown to relieve stress. Indeed, daily intake of live CP2305 for four weeks ameliorated stress-associated symptoms in medical students participating in a cadaver dissection course [16] and improved the clinical symptoms of patients with IBS [17]. Heat-inactivated CP2305 exerted similar stress-relieving effects when tested using the same cadaver dissection stress [18]. Further, the daily intake of a heat-inactivated, washed CP2305-containing beverage for 12 weeks significantly reduced stress-associated mental and physical symptoms, sleep disturbance, and elevation of stress-responsive microRNAs in peripheral blood associated with preparation for a national certification examination [19].

Formulation of heat-inactivated bacteria in tablets has the following advantages: preservation, portability, less sugar, and fewer artificial ingredients. Tablets containing heat-inactivated CP2305 do not include any bioactive metabolites produced by live bacteria. However, heat-inactivated CP2305 itself exhibited bioactivity in our previous studies [16,17,18,19]. Based on these observations, this study was designed to evaluate a tablet containing heat-inactivated, washed, and dried CP2305 as a stress-relieving para-psychobiotic, using the national examination stress model. We used psychological questionnaire scores and biological stress responses based on salivary cortisol and chromogranin A levels as the primary outcomes, and sleep electroencephalogram (EEG) and changes in the gut microbiome as the secondary outcomes.

## 2. Materials and Methods

### 2.1. Participants and Study Design

The present clinical trial was approved by the Institutional Review Board of Tokushima University Hospital and was conducted according to the ethical standards established in the 1964 Declaration of Helsinki. This study was registered with the University Hospital Medical Information Network (UMIN) Clinical Trials Registry as “Research on a lactic acid bacterium preparation for stress relief” (UMIN000027303).

The study was designed as a double-blind, placebo-controlled, parallel-group clinical trial and ran from July 2017 to March 2018 (Table 1). Sample size calculation was performed with G*Power 3.1 [20] using *F*-test between factors with two groups (CP2305 and placebo) and three evaluation sessions (before, 12, and 24 weeks after administration). Assuming an a priori effect size of 0.25 estimated from our previous study [19], an α error probability of 0.05, and a power (1-β error probability) of 0.80, the resulting total sample size is 68. Seventy-four sixth-grade medical students were recruited at Tokushima University, Tokushima, Japan. Written informed consent was obtained from all participants, and they were randomly allocated to either the CP2305 or placebo group with a stratified randomization by gender. Seven of the 74 participants were excluded as they were taking medications that could affect sleep quality; three females were excluded for hormonal contraceptives; and four others were excluded owing to active disease (mental disease, inflammatory disease, bone disease, and hormonal disorder). BMI, alcohol consumption, and smoking were not exclusion criteria. Finally, 29 participants (20 males and nine females) were allocated to the CP2305 group and 31 (21 males and 10 females) to the placebo group. All participants had no history of taking medications within the three months prior to study enrollment or during the study period; none of the participants had a history of psychiatric or other disease. Females were not taking hormonal contraceptives. The participants were instructed to ingest two tablets (placebo or CP2305) once daily for 24 weeks. To assess compliance, participants self-recorded tablet intake. During the trial, the participants were asked not to consume fermented milks, foods containing live lactic acid bacteria, or other probiotic or prebiotic products.

### 2.2. Tablets

Both the CP2305-containing and placebo tablets were prepared using the same procedures and formula except for the presence or absence of heat-inactivated, washed, and dried CP2305 (1 × 10^10^ bacterial cells per 2 tablets). The active tablet was composed of maltose, dextrin, starch, heat-inactivated lactic acid bacteria powder, and vegetable oil. The placebo tablet was similarly composed except that the lactic acid bacteria powder was replaced with dextrin. The formula was allergen-free.

### 2.3. Questionnaires to Assess Mental and Physical States

The physical and mental health of the participants was evaluated using the following questionnaires: the Spielberger State-Trait Anxiety Inventory (STAI) [21], the 28-item General Health Questionnaire (GHQ-28) [22], and the Hospital Anxiety and Depression Scale (HADS) [23]. Sleep was assessed using the Pittsburgh Sleep Quality Index (PSQI) [24]. Participants completed the questionnaires three times, two weeks before and 12 or 24 weeks after the intervention. Stress-associated symptoms (mental irritability, abdominal discomfort, feeling tired, and sleep disturbance) were also assessed using a 100 mm visual analog scale (VAS) ranging from none (0 mm) to worst (100 mm). The participants reported their symptoms by marking the VAS every week during the experimental period.

### 2.4. Measurements of Salivary Cortisol and Chromogranin A (CGA)

Saliva was collected within three days before and 12 or 24 weeks after the intervention start between 16:00 and 17:00, to avoid diurnal fluctuations, using Salivette sampling devices (Sarstadt Inc., Rommelsdorf, Germany) as previously described [25]. Concentrations of salivary CgA (YK070 Human CgA EIA kit; Yanaihara Institute, Shizuoka, Japan), cortisol (Expanded Range High Sensitivity Salivary Cortisol Enzyme Immunoassay kit; Salimetrics Inc., LLC, Carlsbad, CA, USA), and protein (Protein Quantification Kit-Wide Range; Dojindo Inc., Kumamoto, Japan) were measured according the manufacturer’s instructions. Saliva samples were stored at −80 °C until analysis.

### 2.5. Measurement and Assessment of Single-Channel Sleep Electroencephalogram (EEG)

A single-channel EEG was used to record brain activity overnight (Sleep Scope^TM^; SleepWell Co., Osaka, Japan). This instrument is approved as medical equipment (Certification No. 27ADBZX00087000) in Japan and has been widely used in sleep studies [26,27]. After the participants were trained, they wore the portable EEG monitor on three separate occasions before and 24 weeks after starting the supplements. All subjects were required not to consume alcohol on the day the EEG was performed. The data from the first measurement was treated as practice. The data collected from the second measurement were analyzed unless data collection was unsuccessful owing to a failure in data gathering, alcohol intake, or a lack of sufficient sleep-associated data. In those cases, the third record was used for analysis. The sleep stages were scored in accordance with the American Academy of Sleep Medicine Manual [28].

### 2.6. Assessment of Stool Properties and Bowel Habits

All participants recorded the frequency of their bowel movements and fecal characteristics (form, color tone, and output volume) seven consecutive days before and 12 or 24 weeks after the intervention began. Fecal form and color tone were assessed using the Bristol Stool Scale [29]. The fecal output volume was measured using a circular cylinder with a base diameter of 2.5 cm and a length of 5.0 cm.

### 2.7. Measurement of Short-Chain Fatty Acid (SCFA) Concentrations in Feces

Fecal samples were collected within three days before the start of tablet intake and at the end of the study. Concentrations of acetic acid, propionic acid, n-butyric acid, isobutyric acid, n-valeric acid, and isovaleric acid were measured according to the method of Ikeda et al. [30] by high-performance liquid chromatography with a pH indicator (LaChrom Elite; Hitachi High-Technologies Corporation, Tokyo, Japan).

### 2.8. Fecal Microbiota Analysis

To analyze the participants’ intestinal microbiota, fecal samples were collected at two time points, within three days before and 24 weeks after the start of the supplementation. The intestinal microbiota analysis was performed by high-throughput sequencing of the 16S rRNA gene using a MiSeq V2 kit (Illumina, San Diego, CA, USA) as described by Hatanaka et al. [31]. Briefly, the fecal samples were washed in phosphate-buffered saline and centrifuged. Pellets were resuspended in 166 mmol L^−1^ Tris–HCl buffer (pH 9.0) containing 66 mmol l^−1^ EDTA, 8.3% sodium dodecyl sulfate, and 66% saturated phenol in Tris–EDTA (TE) buffer (10 mmol l^−1^ Tris–HCl (pH 8.0), 1 mmol l^−1^ EDTA (pH 8.0)). Glass beads (0.1 mm diameter) were added to the suspension, and the mixture was vortexed vigorously for 60 s using a Multi Beads ShockerR (Yasui Kikai Corporation, Osaka, Japan). After centrifugation at 18,700 *g* for 5 min at 4 °C, the supernatant was extracted with phenol–chloroform–isoamyl alcohol (25:24:1), and DNA was precipitated with isopropanol. The precipitates were washed with 70% ethanol and dissolved with TE buffer. For further purification, a High Pure PCR Template Preparation Kit (Roche, Tokyo, Japan) was used according to the manufacturer’s instructions. Gene sequencing of 16S rRNA was performed as described previously [32]. Briefly, the purified DNA was used as the template for amplicon PCR, and the V4 fragment of the 16S rRNA was amplified with a primer set of Tru357F (5’-CGCTCTTCCGATCTCTG TACGGRAGGCAGCAG-3’) and Tru806R (5’-CGCTCTTCCGATCTGAC GGACTACHVGGGTWTCTAAT-3’). After the PCR products were purified by Agencourt AMPure XP (Beckman Coulter, Inc., CA, USA), the products were amplified using the Nextera Index Kit (Illumina, CA, USA). After the second PCR, amplified products were purified using Agencourt AMPure XP. The library was quantified, normalized, and pooled in an equimolar amount. Sequencing was performed with an Illumina MiSeq system and MiSeq Reagent Kit v.2 (300 Cycle). Sequence data were analyzed as described previously [32]. In brief, Quantitative Insights Into Microbial Ecology (QIIME) ver.1.8.0 was used for sequence filtering and analysis. Quality filtering was performed using fastq files, and sequences with a quality score <29 were removed. Chimeric sequences were removed using USEARCH. Assignment to operational taxonomic units (OTUs) was performed using open-reference OTU picking with a 97% threshold for pairwise identity. After removing the OTUs containing <5 sequences, the OTUs were classified taxonomically using the Greengenes reference database (https://greengenes.secondgenome.com/?prefix=downloads/greengenes_database/gg_13_5/). For detecting overall variation of abundant bacteria (relative abundance of genera accounting for >1% of the total of sequences in feces), normalization was not performed to analyze the relative abundance of major genera in feces.

### 2.9. Statistical Analysis

Statistical analysis was performed using JMP v.13.0 (SAS Japan, Tokyo, Japan). The data are presented as the mean ± standard error of the mean (SEM). The time-dependent changes of the questionnaire scores, salivary stress marker levels, and stool property scores of the participants were analyzed by two-way ANOVA with repeated measures. The VAS scores measured were averaged every six weeks, and their changes were analyzed by two-way ANOVA with repeated measures. The changes in sleep EEG parameters, relative abundances of fecal microbiota, and fecal SCFA levels were analyzed by analysis of covariance (ANCOVA) with each initial value as the covariate. Differences were considered significant at *p* < 0.05. Effect size was estimated from partial eta squared, calculated as the ratio of variance associated with an effect plus that effect and its associated error variance.

## 3. Results

### 3.1. Participant Demographics

Participants were randomly assigned to one of two groups: CP2305 (21 males and 10 females) or placebo (20 males and 9 females). As shown in Table 2, there were no significant differences in age, male/female ratio, body mass index (BMI), or STAI state, STAI trait, HADS anxiety, HADS depression, GHQ 28, and PSQI global scores between the two groups. The mean scores of the questionnaires were within the normal limits. The tablet consumption rates were 95.3 ± 1.0 and 92.6 ± 1.6% in the CP2305 and the placebo groups, respectively. No adverse events were observed throughout the trial.

### 3.2. Effects of CP2305 on Mental and Physical Conditions

**Figure 1:**
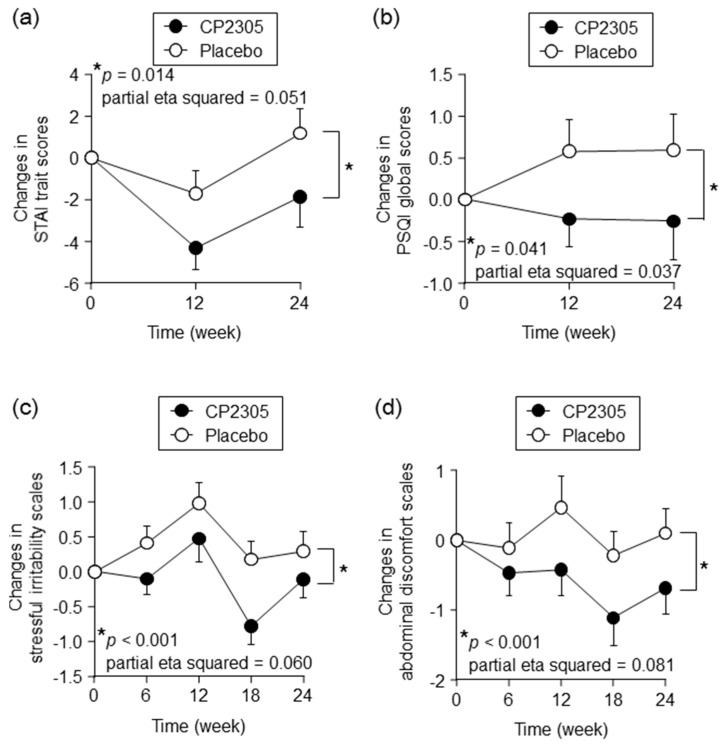
Effects of CP2305 intake on time-dependent changes in questionnaire and visual analog scores. Time-dependent changes in scores of STAI trait anxiety (**a**) and PSQI global score (**b**) in the CP2305 and placebo groups are shown. In addition, the visual analog scales were used to assess stressful irritability (**c**) and abdominal discomfort (**d**). The black circles represent CP2305 and the white represent the placebo groups. Data are presented as the mean ± SEM. The *p* values and partial eta squared values are shown in each panel; these were determined by repeated-measures ANOVA between the groups without multiple comparison correction. Asterisks indicate overall significance between the groups across time points. * *p* < 0.05.

Time-dependent changes in the questionnaire scores are summarized in Table S1. The mean values of the STAI-state scores in the CP2305 and placebo groups were within normal limits before and 12 weeks after starting the interventions, and they were increased above the threshold value of 40 at 24 weeks (two weeks before the examination). However, there was no significant difference in the elevation of STAI-state score between the two groups. Although STAI-trait scores remained within normal limits during the intervention period, CP2305 intake significantly reduced STAI-trait anxiety scores compared to the placebo intake (Figure 1a). The CP2305 supplement also significantly improved sleep quality as assessed by the PSQI questionnaire when compared with the placebo (Figure 1b). There was no significant difference in the global GHQ-28 scores between the two groups, but the CP2305 group documented significantly lower depression scores using the GHQ-28 (Table S1). The HADS questionnaire also show that CP2305 intake ameliorated anxiety and depressive moods relative to placebo (Table S1).

In addition to the above questionnaires, stress-associated symptoms were also evaluated using the VAS, where daily intake of CP2305 significantly improved subjective feelings of irritability (Figure 1c) and abdominal discomfort (Figure 1d).

### 3.3. Effects of CP2305 on Sleep

The quality of sleep was assessed using a single-channel sleep EEG, and the results are summarized in Table 3. There was no significant time-dependent change nor group difference in total rapid eye movement (REM) and non-REM sleep times (Table 3). Delta power (high-amplitude slow wave: 0.5–2.0 Hz, 75 μV) is an indicator of deep sleep observed during the N3 stages of non-REM sleep, a period also referred to as slow wave sleep (SWS). Although there were no significant time-dependent changes nor group differences in total delta power, the intake of CP2305-containing tablets significantly increased the ratio of EEG delta power in the first sleep cycle, compared to placebo intake. In addition, the CP2305 intake significantly shortened the sleep latency of the first N3 stage and wake time after sleep onset compared to placebo intake (Table 3). Thus, CP2305 seemed to improve the quality of sleep in participants experiencing chronic stress.

### 3.4. Effects of CP2305 on Salivary Stress Markers

**Figure 2:**
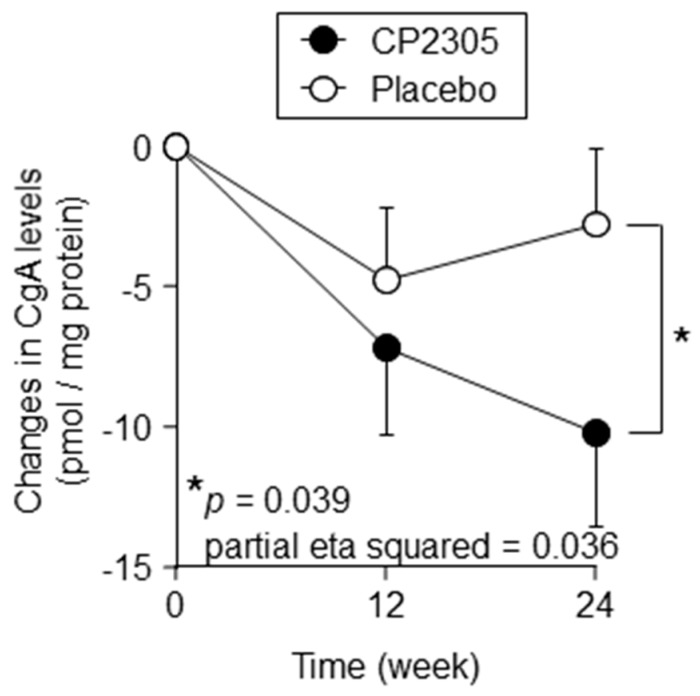
Effects of CP2305 on time-dependent changes in salivary chromogranin A (CgA) levels. Saliva was collected at a constant time within 3 days before and 12 or 24 weeks after the intervention start. Data are presented as the mean ± SEM. Black circles are indicative of CP2305 and the white circles of placebo. Data were analyzed by repeated-measures ANOVA between the groups without multiple comparison correction. *p* and partial eta squared values are shown in the panel. Asterisks indicate overall significance between the groups across time points. * *p* < 0.05.

The stress response during the preparative period for the national examination was also evaluated by measuring salivary CgA and cortisol levels. Salivary CgA levels were significantly decreased in the CP2305 group relative to those of the placebo group (Figure 2 and Table S2), whereas there was no significant difference in salivary cortisol levels between the two groups during the intervention period (Table S2).

### 3.5. Effect of CP2305 on Bowel Habits

**Figure 3:**
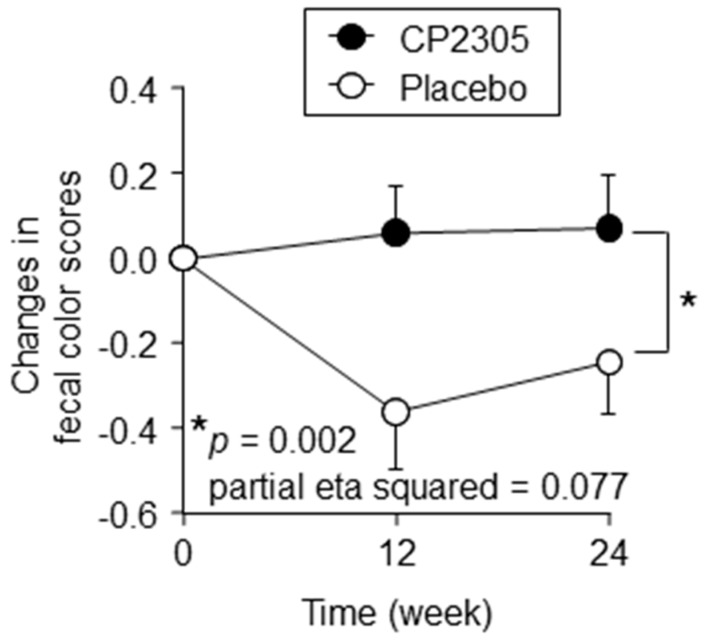
Effects of CP2305 on the time-dependent changes in fecal color. Fecal color tone was assessed using the Bristol Stool Scale. Black circles represent data from participants receiving CP2305 and white circles placebo. Data are presented as the mean ± SEM. Data were analyzed by repeated-measures ANOVA between the groups without multiple comparison correction. *p* and partial eta squared value are shown in the panel. Asterisks indicate overall significance between the groups across time points. * *p* < 0.05.

As shown in Table S3, there were no significant changes in the frequency of defecation, stool output, and stool form over time or between groups. However, the daily intake of the CP2305 tablets significantly changed the color tone relative to placebo, wherein the stool was lightened by CP2305 intake (Figure 3).

### 3.6. Effects of CP2305 on Fecal Microbiota and SCFAs in Feces

The stress-induced changes in the fecal microbiota and modification of the bacterial communities with the daily CP2305 intake were examined at the genus level using next-generation sequencing. The results are shown in Table 4. Notably, the stress encountered during preparations for the national examination decreased the relative abundance of *Bifidobacterium* and increased that of *Streptococcus* in the placebo group. The daily intake of CP2305 significantly mitigated the reduction in *Bifidobacterium* and prevented the elevation of *Streptococcus* (Table 4).

The effect of daily CP2305 intake on the concentrations of SCFAs (acetic acid, propionic acid, n-butyric acid, isobutyric acid, n-valeric acid, and isovaleric acid) in feces was also tested (Table 5). Among the SCFAs measured, only n-valeric acid concentrations were significantly increased in the CP2305 group relative to the placebo group.

## 4. Discussion

This study aimed to assess whether long-term use of a tablet containing heat-inactivated, washed, and dried *Lactobacillus gasseri* CP2305 would have health benefits in young adults preparing for the national examination for medical practitioners. This model has been used in multiple studies of chronic psychological stress and is considered appropriate [25,33]. Of note, our research group used this model to study the effects of daily intake of a beverage containing heat-inactivated, washed *Lactobacillus gasseri* CP2305 for 12 weeks on stress-associated symptoms [19]. Although the stress-associated symptoms were less remarkable in the study presented here, the experimental period was longer (12 vs. 24 weeks), and the present study confirms that the regular intake of a tablet containing heat-inactivated CP2305 improves stress-associated symptoms. Particularly, the CP2305 tablet reduced the trait anxiety score of STAI, which was not detected in shorter studies [16,17,18,19]. This study did not document CP2305-associated suppression of basal salivary cortisol levels, which was documented with the use of the CP2305 beverage [19]. Salivary cortisol levels may be influenced by the menstrual cycle in women. Further analysis of the results that were different among men and women showed no significant effect on stress-induced salivary cortisol levels between the sexes (Table S4). However, the CP2305 tablet significantly reduced salivary CgA levels.

More importantly, these results confirm the significant improvement of sleep quality with the administration of a CP2305 tablet, which was also demonstrated by the CP2305 beverage [19]. Psychological stressors prolong sleep latency and reduce delta power preferentially in the first sleep cycle [34]. In addition to the improvement of sleep quality subjectively assessed by PSQI, a single-channel sleep EEG demonstrated that the daily intake of the CP2305 tablet significantly shortened the sleep latency to the N3-stage and decreased total wake time after sleep onset. Concerning delta power, the CP2305 tablet intake significantly increased the delta power ratio in the first non-REM sleep period compared with the placebo tablet. SWS in the first sleep cycle is particularly important for physiological functions. For instance, growth hormone (GH) is preferentially released during this stage [35]. Similarly, live *Lactobacillus casei* strain Shirota ameliorated academic stress-induced sleep disturbance in healthy adults in association with an increase in delta power [36]. These data suggest that distinct *Lactobacillus* strains may improve the quality of sleep during stressful situations. Some reports suggest a negative correlation between trait anxiety and sleep quality. Indeed, in our subjects, a positive correlation between PSQI and STAI-trait scores (Pearson’s r = 0.3138, *p* < 0.001) was observed. Although the precise mechanism remains unknown, long-term administration of CP2305 may improve sleep quality and result in alteration of the anxiety trait.

Unfortunately, earlier studies did not assess changes to the gut microbiota while taking heat-inactivated, washed CP2305. Given that stress has been reported to change the fecal microbiota composition in animal [37,38,39] and human studies [40,41], this parameter was included in the present study. Indeed, the daily intake of the CP2305 tablet significantly prevented the stress-induced reduction in the relative abundance of *Bifidobacterium* in feces, which was observed in the placebo group. *Bifidobacterium* have been linked to improvements in the intestinal environment by regulating immunological responses, preventing infection, reducing pathogenic bacteria, and producing health-promoting metabolites, thus exerting beneficial effects on allergies, inflammatory bowel disease, IBS, and cancer [42,43]. Of note, Logan and Katzman [44] reported that emotional stress leads to acute and long-term reductions in *Bifidobacterium*, suggesting the high sensitivity of this genus to emotional stress. Additionally, the administration of *Bifidobacterium* reduces anxiety and depression-like behaviors while suppressing peripheral proinflammatory cytokines and increasing plasma tryptophan, which have been implicated in depression in animal models [45]. The preservation of a high abundance of *Bifidobacterium* in the microbiota may improve stress-associated mental and physical symptoms. The CP2305 tablet also prevented the increase in the relative abundance of *Streptococcus*, which was noted in the placebo. This is supported by work from Suzuki et al. [37], who found that the abundance of *Streptococcus* was significantly increased in rats exposed to crowding stress. Such an increase in *Streptococcus* may be considered harmful in humans, as streptococci are associated with increased risk of colorectal cancer [46].

Previous studies have also demonstrated that some probiotics alter the gut microbiota production of SCFAs [47,48,49]. The production of SCFAs can stimulate colonocytes, including endocrine epithelial cells, and gut hormones, subsequently contributing to gut–brain axis activation [50,51]. Thus, the concentrations of major SCFAs in feces were measured; the concentrations of n-valeric acid were significantly increased in participants that received CP2305 during the intervention period compared to placebo. Yuille et al. [52] have shown that n-valeric acid is a potent inhibitor of class-I histone deacetylase (HDAC) using HT-29 human colon cancer cells. The concentration of n-valeric acid in Yuille’s study (approximately 50 mM) was higher than that found in the feces in the current study (approximately 10–15 mM). The physiological significance of n-valeric acid in the feces is not fully understood. Further studies on this point are needed. A significant improvement in fecal color tone was also observed in response to CP2305 tablet intake, which is suggestive of more acidic conditions. Thus, the long-term consumption of CP2305 likely improves the intestinal environment even under stressful situations and ameliorates stress-associated symptoms.

Our study has some important limitations. No dietary data were collected to determine whether dietary habits in both groups influenced changes in the n-valeric acid concentration in the stool. No information regarding the menstrual cycle was obtained from the women participants, which may influence salivary cortisol levels. 

The mechanism underlying the stress-relieving effects of *Lactobacillus gasseri* CP2305 is unknown. However, the strain is known to colonize the intestines, and it has been observed that heat-inactivated cells can stimulate the afferent vagal nerve when administered to rat stomach or intestine (unpublished observations). Thus, these features may effectively stimulate the gut–brain axis directly or indirectly and may modify HPA axis activity, resulting in the improvement of stress-associated symptoms and the intestinal environment. However, this remains to be proven. Regardless, the present study suggests that a tablet containing heat-inactivated, washed, and dried *Lactobacillus gasseri* CP2305 may be beneficial for young adults experiencing stressful conditions. Moreover, the development of a tablet containing CP2305 widens the commercial applications of this bacterium.

## Figures and Tables

**Table 1:** Experimental schedule.

Measurements/Events	Period of Time (Weeks)				
	−2	0	12	24	26
Tablet intake		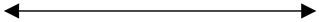			
Questionnaires	●		●	●	
Weekly diary	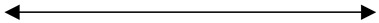				
Saliva sampling (1 day)	●		●	●	
EEG waves measurement (3 days)	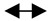			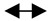	
Fecal sampling (3 days)	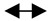			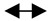	
Defecation diary (7 days)	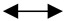		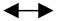	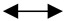	
National examination (2 days)					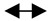 *

●: Spot event, 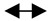: Once a day in the indicated period * All subjects took the national examination for medical practitioners. EEG: electroencephalogram.

**Table 2:** Comparison of baseline characteristics between CP2305 and placebo groups.

Parameters	CP2305	Placebo	*p* Value
Age (years)	24.9 ± 0.5	25.3 ± 0.6	0.54
Sex (male/female)	21/10	20/9	0.91
BMI (kg/m^2^)	21.1 ± 0.5	20.8 ± 0.5	0.65
STAI state anxiety	37.1 ± 1.6	35.8 ± 1.6	0.58
STAI trait anxiety	41.8 ± 1.7	39.7 ± 1.8	0.40
HADS anxiety	4.8 ± 0.5	4.3 ± 0.5	0.45
HADS depression	5.8 ± 0.5	5.2 ± 0.5	0.43
GHQ-28 total	4.1 ± 0.6	3.7 ± 0.6	0.64
PSQI global score	4.2 ± 0.4	3.4 ± 0.4	0.16
% of daily test tablet consumption	95.3 ± 1.0	92.6 ± 1.6	0.17

Data are presented as mean ± standard error of the mean (SEM). Data were analyzed by the Student’s *t*-test. The χ^2^ test was used for analysis of the male/female ratio. BMI: body mass index; STAI: Spielberger State-Trait Anxiety Inventory; HADS: Hospital Anxiety and Depression Scale; GHQ-28: 28-item General Health Questionnaire; PSQI: Pittsburgh Sleep Quality Index.

**Table 3:** Sleep measurements.

Parameters	Treatment	Values *		
		Week 0	Week 24	Changes
Total N3 stage (min)	CP2305	41.6 ± 5.0	46.1 ± 5.3	4.5 ± 3.4
	Placebo	43.9 ± 4.9	46.1 ± 5.2	2.2 ± 3.3
Total REM sleep time (min)	CP2305	93.4 ± 5.7	84.8 ± 5.7	−8.5 ± 5.5
	Placebo	79.4 ± 5.6	81.8 ± 5.6	2.4 ± 5.4
Delta power in total sleep period time (μV2)	CP2305	520,644 ± 61,030	483,302 ± 54,273	−37,342 ± 25,529
	Placebo	482,451 ± 59,889	456,930 ± 53,259	−25,522 ± 25,052
Delta power ratio (%) ^†,‡^	CP2305	37.2 ± 2.7	50.4 ± 2.9	13.2 ± 2.9
	Placebo	41.2 ± 2.6	44.4 ± 2.9	3.2 ± 2.8
Sleep latency of first N3 stage (min) ^†^	CP2305	23.7 ± 3.6	17.6 ± 5.6	−6.0 ± 5.4
	Placebo	22.0 ± 3.5	26.3 ± 5.5	4.3 ± 5.3
Wake time after sleep onset (min) ^†^	CP2305	22.7 ± 2.1	18.4 ± 2.1	−4.2 ± 2.0
	Placebo	22.7 ± 1.0	21.8 ± 2.1	−0.9 ± 1.9

* Data are presented as the mean ± SEM. ^†^ Significant differences between CP2305 and placebo group (*p* < 0.05) by analysis of covariance (ANCOVA) with each initial value as a covariate. Partial eta squared values of delta power ratio, sleep latency of first N3 stage, and wake time after sleep onset are 0.099, 0.085, and 0.120, respectively. ^‡^ Delta power ratio is representative of the delta power in the first sleep cycle relative to the delta power in the total sleep period. Abbreviations: REM, rapid eye movement; N3, non-REM sleep stage 3.

**Table 4:** Relative abundance of major genera within the fecal microbiota.

Genus	Treatment	Composition of Bacterial Genus (%) *		
		Baseline	24 Weeks	Change
*Bifidobacterium* ^†^	CP2305	9.8 ± 1.8	6.1 ± 1.3	−3.6 ± 1.2
	Placebo	14.7 ± 2.6	6.8 ± 1.3	−7.9 ± 2.3
*Faecalibacterium*	CP2305	10.5 ± 1.8	16.5 ± 2.0	6.0 ± 1.4
	Placebo	6.8 ± 1.0	12.4 ± 1.9	5.6 ± 1.7
*Roseburia*	CP2305	4.5 ± 1.2	4.7 ± 1.0	0.2 ± 1.2
	Placebo	2.8 ± 0.6	6.6 ± 1.5	3.7 ± 1.4
*Streptococcus* ^†^	CP2305	1.8 ± 0.7	1.6 ± 0.6	−0.2 ± 0.3
	Placebo	1.3 ± 0.4	1.8 ± 0.5	0.5 ± 0.6
*Dorea*	CP2305	1.5 ± 0.2	1.8 ± 0.3	0.3 ± 0.3
	Placebo	1.7 ± 0.2	1.9 ± 0.4	0.2 ± 0.4
*Lachnospiraceae; Other* ^†^	CP2305	2.1 ± 0.4	1.6 ± 0.3	−0.5 ± 0.2
	Placebo	1.4 ± 0.3	1.4 ± 0.2	0.0 ± 0.2
*Ruminococcus*	CP2305	1.7 ± 0.4	2.9 ± 0.6	1.2 ± 0.7
	Placebo	1.2 ± 0.3	2.8 ± 0.8	1.6 ± 0.8

* Data are presented as the mean ± SEM. ^†^ Significant differences between CP2305 and placebo groups (*p* < 0.05) by ANCOVA with each initial value as a covariate. Partial eta squared values of *Bifidobacterium*, *Streptococcus*, and *Lachnospiraceae; Other* are 0.096, 0.152, and 0.158, respectively.

**Table 5:** Concentration of short-chain fatty acids (SCFAs) in feces.

SCFAs	Treatment	Values * (mg g^−1^ feces)		
		Week 0	Week 24	Change
Acetic acid	CP2305	38.3 ± 2.4	32.0 ± 1.9	−6.3 ± 2.7
	Placebo	38.5 ± 2.6	30.5 ± 2.0	−8.0 ± 2.8
Propionic acid	CP2305	13.0 ± 1.1	12.9 ± 0.9	−0.1 ± 1.2
	Placebo	12.6 ± 1.1	11.8 ± 0.9	−0.8 ± 1.3
n-Butyric acid	CP2305	8.5 ± 1.1	9.3 ± 1.1	0.8 ± 1.3
	Placebo	8.6 ± 1.1	7.8 ± 1.1	−0.9 ± 1.3
iso-Butyric acid	CP2305	0.9 ± 0.1	1.4 ± 0.2	0.5 ± 0.2
	Placebo	1.1 ± 0.1	1.0 ± 0.2	−0.1 ± 0.2
n-Valeric acid ^†^	CP2305	1.2 ± 0.2	1.6 ± 0.2	0.4 ± 0.2
	Placebo	1.5 ± 0.2	1.0 ± 0.3	−0.6 ± 0.2
iso-Valeric acid	CP2305	1.3 ± 0.2	1.9 ± 0.3	0.6 ± 0.3
	Placebo	1.6 ± 0.2	1.4 ± 0.3	−0.2 ± 0.3

* Data are presented as the mean ± SEM. ^†^ Significant differences between CP2305 and placebo group (*p* < 0.05) by ANCOVA with each initial value as a covariate. Partial eta squared value of n-valeric acid is 0.114.

## Supplementary Materials

The following are available online at https://www.mdpi.com/2072-6643/11/8/1859/s1, Table S1. Time-dependent changes in questionnaire scores. Table S2. Time-dependent changes in salivary stress markers. Table S3. Time-dependent changes in stool properties and bowel habits, Table S4. Gender-stratified analysis of salivary cortisol.
