# Supplementary material for: Health Benefits of Lactobacillus gasseri CP2305 Tablets in Young Adults Exposed to Chronic Stress: A Randomized, Double-Blind, Placebo-Controlled Study
Source: Nutrients. 2019 Aug 10;11(8):1859. doi: 10.3390/nu11081859 (PMC6723420; doi:10.3390/nu11081859)
Supplement: Supplementary file 1 [file nutrients-11-01859-s001.pdf]

Table S1. Time-dependent changes in questionnaire scores.

Questionnaire	Probability for each factor			Treatment	Scores		
	Treatment	Time	Time x Treatment		Week 0	Week 12	Week 24
STAI state anxiety	0.786	< 0.001	0.670	CP2305	37.1 ± 1.6	34.2 ± 1.5	45.9 ± 1.7
				Placebo	35.8 ± 1.6	35.1 ± 1.9	43.4 ± 2.2
STAI trait anxiety	0.014 *	0.699	0.093	CP2305	41.8 ± 1.8	37.7 ± 1.6	39.9 ± 1.2
				Placebo	39.7 ± 1.8	38.2 ± 1.8	40.8 ± 2.2
HADS anxiety	0.072	0.007	0.397	CP2305	4.8 ± 0.5	4.6 ± 0.5	5.5 ± 0.6
				Placebo	4.3 ± 0.5	5.4 ± 0.7	5.6 ± 0.7
HADS depression	0.071	0.003	0.800	CP2305	5.8 ± 0.5	5.0 ± 0.6	7.0 ± 0.8
				Placebo	5.2 ± 0.6	6.3 ± 0.8	6.7 ± 0.7
GHQ-28 total	0.615	0.013	0.667	CP2305	4.1 ± 0.7	3.6 ± 0.6	5.0 ± 0.6
				Placebo	3.7 ± 0.6	3.1 ± 0.6	4.9 ± 0.8
GHQ-28 depression	0.022 *	0.545	0.249	CP2305	0.06 ± 0.04	0.00 ± 0.00	0.03 ± 0.03
				Placebo	0.07 ± 0.07	0.22 ± 0.12	0.66 ± 0.12
PSQI global score	0.041 *	0.542	0.143	CP2305	4.2 ± 0.4	4.1 ± 0.5	3.9 ± 0.4
				Placebo	3.4 ± 0.3	3.9 ± 0.4	4.1 ± 0.5

Data are shown as the means ± SEM.

*Significant different between CP2305 and placebo groups ($p < 0.05$ by repeated-measures ANOVA).

Table S2. Time-dependent changes in salivary stress markers.

Parameters	Probability for each factor			Treatment	Values		
	Treatment	Time	Time x Treatment		Week 0	Week 12	Week 24
Chromogranin A	0.039 *	0.001	0.056	CP2305	21.0 ± 2.8	13.8 ± 2.2	10.8 ± 2.1
(pmol/mg protein)				Placebo	15.2 ± 2.9	10.5 ± 2.2	12.5 ± 2.2
cortisol (μg/dL)	0.086	< 0.001	0.716	CP2305	0.14 ± 0.02	0.28 ± 0.03	0.28 ± 0.03
				Placebo	0.14 ± 0.02	0.18 ± 0.03	0.26 ± 0.03

Data are shown as the means ± SEM. *Significant different between CP2305 and placebo groups ($p < 0.05$ by repeated-measures ANOVA).

Table S3. Time-dependent changes in stool properties and bowel habits.

Questionnaire	Probability for each factor			Treatment	Scores		
	Treatment	Time	Time x Treatment		Week 0	Week 12	Week 24
Frequency of bowel movement	0.965	0.533	0.987	CP2305	9.6 ± 0.7	9.5 ± 0.8	9.4 ± 1.0
				Placebo	9.2 ± 0.8	9.2 ± 1.1	9.1 ± 0.7
Output	0.677	0.356	0.833	CP2305	25.3 ± 2.4	25.4 ± 2.3	24.2 ± 2.1
				Placebo	26.0 ± 3.1	25.0 ± 4.0	24.6 ± 2.5
Form	0.546	0.011	0.791	CP2305	4.0 ± 0.1	4.0 ± 0.1	3.7 ± 0.1
				Placebo	4.0 ± 0.1	3.6 ± 0.2	3.7 ± 0.2
Color	0.002 *	0.320	0.079	CP2305	3.5 ± 0.1	3.6 ± 0.2	3.6 ± 0.1
				Placebo	3.6 ± 0.1	3.2 ± 0.1	3.3 ± 0.1

Data are shown the means ± SEM.

*Significant different between CP2305 and placebo groups ($p < 0.05$ by repeated-measures ANOVA).

Table S4. Gender-stratified analysis of salivary cortisol.

Parameters	Probability for each factor			Treatment	Values (μg/dL)		
	Treatment	Time	Time x Treatment		Week 0	Week 12	Week 24
Women	0.251	0.016	0.645	CP2305	0.14 ± 0.03	0.26 ± 0.06	0.29 ± 0.08
				Placebo	0.11 ± 0.02	0.13 ± 0.02	0.21 ± 0.05
Men	0.208	< 0.001	0.926	CP2305	0.14 ± 0.02	0.28 ± 0.04	0.28 ± 0.04
				Placebo	0.15 ± 0.03	0.21 ± 0.03	0.28 ± 0.03

Data are shown as the means ± SEM. Probability for each factor was analyzed using two-way ANOVA with repeated measures.
